# Association Between the 6-Min Walk Test and Cardiopulmonary Exercise Testing Parameters in Subacute Cerebral Infarction

**DOI:** 10.3390/brainsci16080819

**Published:** 2026-07-31

**Authors:** Jingyuan Qie, Xiaoxi Zhao, Yaqin Gu, Jubao Du, Xin Ma

**Affiliations:** 1 Department of Neurology, Xuanwu Hospital, Capital Medical University, Beijing 100053, China; jing_yuan_qie@yeah.net (J.Q.); zhaoxxmy@163.com (X.Z.); guyq1999@163.com (Y.G.); 2 National Clinical Research Center for Geriatric Disorders, Beijing 100053, China; 3 Department of Neurorehabilitation, Beijing Bo’ai Hospital, China Rehabilitation Research Center, Beijing 100068, China; 4 Department of Rehabilitation Medicine, Xuanwu Hospital, Capital Medical University, Beijing 100053, China; dujubaofei@aliyun.com

**Keywords:** subacute cerebral infarction, 6-min walk test, cardiopulmonary exercise testing, peak oxygen uptake, peak heart rate, cardiorespiratory fitness

## Abstract

**Highlights:**

**What are the main findings?**

- In patients with subacute cerebral infarction, 6-min walk distance was independently associated with CPET-derived peak oxygen uptake.
- CPET-derived peak heart rate was associated with 6MWT-derived parameters, including walking distance, 6MWT peak heart rate, and Borg RPE score.

**What are the implications of the main findings?**

- The 6MWT may provide practical supplementary information related to cardiorespiratory fitness and heart-rate response when CPET is not readily available.
- These findings support the clinical value of 6MWT-derived parameters in stroke rehabilitation assessment.

**Abstract:**

**Background:** Peak oxygen uptake (VO_2_peak), as measured by cardiopulmonary exercise testing (CPET), is considered the gold standard for assessing cardiorespiratory fitness (CRF). However, CPET is not always feasible in rehabilitation settings. The 6-min walk test (6MWT) is simple and widely used, yet its relationship with CPET-derived measures in patients with subacute cerebral infarction remains unclear. **Methods:** A total of 122 patients with subacute cerebral infarction underwent both the 6MWT and CPET. VO_2_peak, CPET-derived peak heart rate (CPET HRpeak), 6-min walk distance (6MWD), 6MWTpeak heart rate (6MWT HRpeak), and the ratio of 6MWT HRpeak to age-predicted maximal heart rate were assessed. **Results:** In multivariate linear regression analysis, 6MWD was independently associated with VO_2_peak (unstandardized coefficient B = 0.010, *p* < 0.001). Partial correlation analyses adjusting for age, sex, and β-blocker use showed that CPET HRpeak was positively correlated with 6MWD (ρ = 0.361, *p* < 0.001) and 6MWT HRpeak (ρ = 0.217, *p* = 0.018). The ratio of 6MWT HRpeak to age-predicted maximal heart rate did not mediate the association between 6MWD and VO_2_peak. **Conclusions:** In patients with subacute cerebral infarction, 6MWD was independently associated with VO_2_peak, while both 6MWD and 6MWT HRpeak were positively correlated with CPET HRpeak. These findings suggest that the 6MWT may help assess CRF when CPET is not available.

## 1. Introduction

Cardiorespiratory fitness (CRF) refers to the capacity of the cardiopulmonary system to deliver oxygen to skeletal muscles during physical exertion. It reflects the integrated physiological performance of multiple systems, including the cardiovascular, respiratory, musculoskeletal, and neurological systems, during exercise. In clinical settings, CRF is commonly quantified by peak oxygen consumption (VO_2_peak) [1]. Reduced CRF after experiencing a stroke is a common and persistent condition; previous studies have reported that the CRF of patients recovering from a stroke is approximately 53% of that observed in healthy individuals [2]. Impaired CRF not only indicates reduced physical activity capacity, but is also closely associated with an increased risk of cardiovascular and cerebrovascular events, including recurrent stroke [3]. Although the first three months after the onset of stroke are generally considered a critical period for functional recovery, and patients during this stage may exhibit gradual improvements in CRF [2], the magnitude of spontaneous recovery frequently remains limited. Therefore, accurate assessment of CRF during this crucial recovery phase, together with the implementation of individualized rehabilitation strategies, is of substantial clinical importance for improving post-stroke outcomes. Cardiopulmonary exercise testing (CPET) is regarded as the gold standard for CRF assessment. Through symptom-limited maximal exercise evaluation, CPET provides parameters such as VO_2_peak and anaerobic threshold (AT), which reflect cardiopulmonary function and facilitate the evaluation of rehabilitation efficacy. Furthermore, additional variables, including peak heart rate (HRpeak) provide valuable information for developing individualized post-stroke exercise intensity [4]. However, CPET requires specialized facilities, expensive equipment, and professionally trained personnel, thereby limiting its widespread clinical application, particularly in primary healthcare environments. Consequently, simpler and more clinically accessible assessment methods are required. The 6-min walk test (6MWT) is a well-established submaximal field-based walking assessment that reflects functional exercise capacity rather than maximal cardiopulmonary performance, and it has been widely used to evaluate CRF in patients with chronic cardiovascular and pulmonary disorders [4,5]. In recent years, the International Stroke Rehabilitation Consensus has additionally recommended the 6MWT as a core assessment tool for walking endurance in patients with stroke [6]. Nevertheless, because stroke survivors frequently experience mobility impairment and gait dysfunction, the validity of the 6MWT for evaluating CRF in this population remains controversial. Previous investigations have demonstrated low-to-moderate correlations between 6-min walk distance (6MWD) and VO_2_peak [4,7], whereas other studies have suggested that 6MWD is predominantly determined by walking ability rather than CRF itself [8]. Therefore, whether 6MWD can function as a surrogate indicator of VO_2_peak requires further investigation. Furthermore, even if such an association exists, the underlying mechanism linking 6MWD and VO_2_peak remains insufficiently understood.

An important objective of CRF assessment in patients with stroke is to inform the assessment of exercise intensity during rehabilitation. Exercise intensity is commonly expressed on the basis of heart rate, and target heart rate is frequently calculated using the Karvonen formula, for which HRpeak is an important reference parameter [4]. The most reliable method for determining HRpeak is CPET. However, it remains unclear whether HRpeak obtained during CPET (CPET HRpeak) in patients with subacute cerebral infarction is associated with heart rate parameters derived from the 6MWT or with walking distance achieved during the test. Examining these associations may provide useful information on how 6MWT-derived parameters relate to CPET HRpeak in the context of assessing exercise intensity based on heart rate.

According to the Fick principle, VO_2_peak is jointly determined by cardiac output and the arteriovenous oxygen difference [9], with heart rate representing a major determinant of cardiac output. Chronotropic competence refers to the capacity of the heart to appropriately increase its rate in response to increasing exercise intensity [10]. In patients with stroke, the association between 6MWD and VO_2_peak may also be affected by factors other than the heart-rate response, such as walking efficiency and walking-related energy expenditure, paretic-side muscle function, balance and motor control, and peripheral oxygen extraction capacity [11,12]. Because heart rate contributes directly to cardiac output, the present study examined the heart-rate response during the 6MWT. The ratio of HRpeak achieved during the 6MWT to age-predicted maximal heart rate (hereafter referred to as 6MWT HRpeak/HRmax-pred) reflects the chronotropic response during walking exercise. Theoretically, patients capable of walking longer distances may exhibit a stronger chronotropic response during the 6MWT, which could subsequently contribute to higher VO_2_peak values. Therefore, 6MWT HRpeak/HRmax-pred may potentially mediate the relationship between 6MWD and VO_2_peak. However, no previous investigation has directly evaluated this mediating pathway. Based on this knowledge gap, we explored whether 6MWT HRpeak/HRmax-pred mediates the association between 6MWD and VO_2_peak in patients with subacute cerebral infarction.

On the basis of these considerations, it was hypothesized that 6MWD would be independently associated with CPET-derived VO_2_peak in patients with subacute cerebral infarction. The objectives of the present study were as follows: (1) to determine whether 6MWD is independently associated with VO_2_peak as measured by CPET in patients with subacute cerebral infarction and to investigate the mediating role of 6MWT HRpeak/HRmax-pred in the association between 6MWD and VO_2_peak; and (2) to examine the correlations between 6MWT parameters and CPET HRpeak.

## 2. Methods

### 2.1. Study Design

This cross-sectional study was approved by the institutional ethics committee. Written informed consent was obtained from all participants before study enrollment. The study was conducted in accordance with the Strengthening the Reporting of Observational Studies in Epidemiology (STROBE) statement [13].

### 2.2. Participants

Patients with cerebral infarction were recruited from the Cardio-Cerebrovascular Center of the Department of Neurology at Xuanwu Hospital and the Department of Neurological Rehabilitation at Beijing Bo’ai Hospital, China Rehabilitation Research Center, between July 2023 and July 2025. The inclusion criteria were as follows: (1) age between 18 and 80 years; (2) duration from stroke onset ranging from 14 days to 3 months; (3) Functional Ambulation Category (FAC) grade 3–5, indicating the capacity to walk for at least 6 min without physical assistance; and (4) provision of signed informed consent. The exclusion criteria included: (1) a history of chronic heart failure, chronic obstructive pulmonary disease, cor pulmonale, or asthma; (2) severe organ failure or malignant disorders such as cancer; (3) the presence of any contraindication to CPET according to the 2022 Chinese Expert Consensus on Clinical Standard Application of Cardiopulmonary Exercise Testing [14]; and (4) psychiatric disorders or cognitive impairment that prevented cooperation with the assessments.

### 2.3. Baseline Characteristics and Functional Evaluation

Patient information was collected through structured interviews, including age, body mass index (BMI), and medical history. Medical history variables included hypertension, diabetes mellitus, hyperlipidemia, coronary heart disease, hyperhomocysteinemia, previous stroke, smoking history, and beta-blocker use.

The Saltin–Grimby Physical Activity Level Scale (SGPALS) was applied to evaluate habitual physical activity intensity before stroke onset. The scale categorizes physical activity into four levels: Level 1, sedentary lifestyle with physical inactivity; Level 2, light physical activity, defined as at least 4 h per week of mild exercise such as cycling or walking for transportation, walking with family members, fishing, or table tennis; Level 3, moderate physical activity, defined as regular exercise or training for at least 2–3 h weekly, including moderate-intensity sports such as running, swimming, tennis, or badminton; and Level 4, vigorous physical activity, defined as participation in competitive sports or intensive training several times per week, including football, basketball, or cross-country activities [15].

The National Institutes of Health Stroke Scale (NIHSS) was employed to quantify stroke severity, and the Fugl–Meyer Assessment (FMA), which has a maximum total score of 100 points, was administered to characterize motor impairment [16]. Balance performance was quantified using the Berg Balance Scale, for which the highest attainable score is 56 points [17].

Walking ability was assessed using the FAC scale. FAC level 3 indicates the ability to walk on level surfaces without direct physical assistance, although supervision or verbal cueing is required for safety. FAC level 4 indicates independent ambulation on level ground, with supervision or physical assistance required for stairs, slopes, or uneven surfaces. FAC level 5 represents complete independent ambulation under all environmental conditions [18].

### 2.4. CPET Testing and Parameters

CPET was performed using an Ergoselect 100 cycle ergometer (Ergoline, Bitz, Germany). Breath-by-breath respiratory data and electrocardiographic signals were collected and analyzed using the MasterScreen-CPX system (JAEGER, CareFusion GmbH, Hoechberg, Germany). Participants were instructed to fast for at least 1.5 h before testing and to avoid caffeine-containing or carbonated beverages on the testing day. The testing procedure followed the standards established by the Harbor-UCLA Medical Center CPET laboratory and employed a continuous incremental ramp protocol to achieve symptom-limited maximal exercise testing. The procedure consisted of a 3-min warm-up period followed by individualized workload increments based on age, sex, height, and body weight. Workload increments of 10, 15, or 20 W/min were applied at 1-min intervals. Participants were instructed to maintain a pedaling frequency of 55–65 rpm throughout the test. After completion of the incremental exercise phase, participants performed unloaded cycling for 3 min, followed by an additional 3-min seated recovery period on the cycle ergometer. The recorded parameters included VO_2_peak, percentage of predicted VO_2_peak, and heart rate at VO_2_peak (CPET HRpeak) [19]. Age-predicted maximal heart rate was calculated using the conventional Fox equation: HRmax-pred = 220 − age [20]. The ratio of CPET HRpeak to age-predicted maximal heart rate was defined as the percentage of HRpeak.

The CPET termination criteria were as follows: (1) persistent myocardial ischemia or severe arrhythmias detected by electrocardiography; (2) symptoms including chest pain, dyspnea, dizziness, muscle weakness, or muscle cramping; (3) a reduction in systolic blood pressure of ≥10 mmHg or persistent systolic blood pressure below baseline values, or systolic blood pressure ≥ 220 mmHg and/or diastolic blood pressure ≥ 110 mmHg; and (4) inability to maintain the required pedaling frequency because of fatigue. The respiratory exchange ratio (RER), defined as the ratio of carbon dioxide production to oxygen consumption, was used to determine whether maximal effort was achieved during CPET. An RER < 1.0 indicated that the measured peak oxygen uptake did not sufficiently represent the participant’s actual CRF [21]; consequently, the corresponding data were excluded from the analysis. CPET was conducted by qualified physicians at both study centers using an identical standardized protocol.

For descriptive purposes, VO_2_peak values were classified according to the Weber classification as follows: class A, no or mild impairment, corresponding to VO_2_peak > 20 mL·kg^−1^·min^−1^; class B, mild-to-moderate impairment, corresponding to 16–20 mL·kg^−1^·min^−1^; class C, moderate-to-severe impairment, corresponding to 10–16 mL·kg^−1^·min^−1^; and class D, severe impairment, corresponding to <10 mL·kg^−1^·min^−1^ [22]. Because these categories were originally established for patients with heart failure, they were applied exclusively as conventional VO_2_peak-based reference ranges and should not be construed as a stroke-specific diagnostic classification.

### 2.5. The 6MWT

Participants wore an HPMS-112 multiparameter monitor (Harbin Boyun Medical Equipment Co., Ltd., Harbin, China) during the assessment. The 6MWT was conducted in accordance with the standardized procedures recommended by the European Respiratory Society and the American Thoracic Society [23]. Participants were instructed to walk back and forth as rapidly as possible along a straight and level 30 m corridor for a duration of 6 min, and the total walking distance was recorded. Resting heart rate, post-test heart rate, blood pressure, respiratory rate, and oxygen saturation were measured using the multiparameter monitoring system. During the 6MWT, heart rate was recorded at 1-min intervals, and the highest recorded value was defined as the 6MWT HRpeak. The ratio of 6MWT HRpeak to age-predicted maximal heart rate (6MWT HRpeak/HRmax-pred) was subsequently calculated. The difference between HRpeak and resting heart rate was defined as heart rate increase (ΔHR). The Borg Rating of Perceived Exertion scale (6–20 points) was used to evaluate the participant’s subjective perception of physical exertion during the 6MWT [24]. The 6MWT and CPET were performed within 3 days of one another, with a median interval of 1.5 days (IQR: 1–2) in the present cohort. Although the test order was randomized, the actual sequence was determined by routine clinical scheduling, patient condition, and equipment availability. On each testing day, the assessment was administered before the scheduled rehabilitation training session to minimize the potential influence of fatigue.

### 2.6. Sample Size Calculation

Sample size estimation was performed using PASS 15 Power Analysis and Sample Size Software (NCSS, LLC, Kaysville, UT, USA). For the first component of the study, the required sample size for multiple linear regression analysis for testing the association between 6MWD and VO_2_peak while allowing for five covariates in the model was estimated based on previous studies [25,26] and clinical relevance. Using the following parameters: Cohen’s f^2^ = 0.15, α = 0.05, statistical power = 90%, and five covariates, the minimum required sample size was calculated as *n* = 70. For the second component of the study, the sample size required for partial correlation analysis was calculated assuming three covariates (age, sex, and β-blocker use), which were selected based on fundamental physiological principles and clinical knowledge. With a moderate partial correlation coefficient of 0.35 (Cohen’s f^2^ = 0.1396), α = 0.05, statistical power = 0.90, and three covariates, the minimum required sample size was *n* = 79. After integrating the two estimations and considering a potential missing or invalid data rate of 15%, the target recruitment sample size was set at a minimum of 93 participants.

### 2.7. Statistical Analysis

All statistical analyses were conducted using R version 4.5.0 (R Core Team, R Foundation for Statistical Computing, Vienna, Austria). Data distribution normality was assessed before analysis, followed by descriptive statistical evaluation. Normally distributed continuous variables were expressed as mean ± standard deviation (SD), whereas non-normally distributed continuous variables were presented as median with interquartile range. Categorical variables were summarized as frequencies and percentages.

To investigate the associations between the 6MWT parameters and VO_2_peak in patients with cerebral infarction, univariate linear regression analyses were initially performed using VO_2_peak as the dependent variable. Variables with *p* < 0.10 in univariate analyses were subsequently included in the multivariable linear regression model, while clinically relevant variables were retained irrespective of statistical significance. A two-sided *p* value < 0.05 was considered statistically significant. Regression assumptions were checked using residual diagnostics, including residual plots and quantile–quantile plots. Model diagnostics focused on the residuals rather than the marginal distribution of VO_2_peak. Multicollinearity was evaluated using the variance inflation factor (VIF), with VIF < 5 considered acceptable.

Subgroup analyses were additionally conducted according to age, sex, walking ability, overweight status, smoking status, regular exercise before the onset of stroke, and medical history to determine whether the observed associations varied across different patient characteristics. Furthermore, partial correlation analyses were performed to evaluate the relationships between CPET HRpeak and the 6MWT parameters after adjustment for relevant confounding variables.

The distribution of variables included in the correlation analyses was assessed before analysis. Pearson or Spearman correlation analyses were used to examine the unadjusted associations between CPET HRpeak and selected 6MWT-related parameters, depending on the data distribution. Partial correlation analyses were further performed after adjusting for age, sex, and β-blocker use, given their potential influence on exercise-related heart-rate responses. Correlation coefficients are reported as Pearson’s r or Spearman’s ρ, as appropriate.

To further examine the association between 6MWD and VO_2_peak, we performed a post hoc exploratory mediation analysis to test whether 6MWT HRpeak/HRmax-pred mediated this relationship. This indirect effect was tested using 5000 bootstrap resamples. Two regression models were fitted. In Model 1, 6MWT HRpeak/HRmax-pred was entered as the dependent variable and 6MWD as the independent variable to estimate path a. In Model 2, VO_2_peak was entered as the dependent variable, with both 6MWD and 6MWT HRpeak/HRmax-pred included as independent variables to estimate path b and the direct effect c′. The mediation model was adjusted for β-blocker use because β-blockers may affect heart-rate responses during exercise. Mediation was considered statistically significant when the 95% bootstrap confidence interval for the indirect effect did not include zero.

## 3. Results

### 3.1. Patient Characteristics

**Figure 1:**
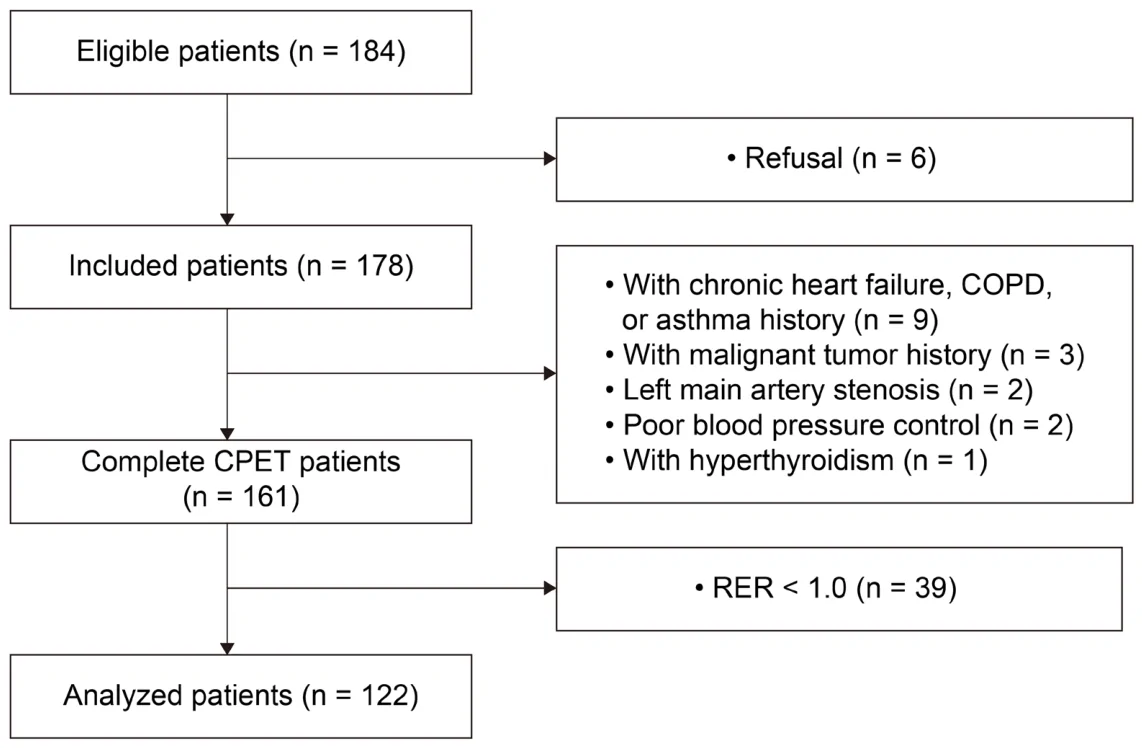
Flow diagram of participant selection. A total of 184 patients were initially screened for eligibility. Following the exclusion process, 122 participants were included in the final analysis. Abbreviations: COPD = chronic obstructive pulmonary disease; CPET = cardiopulmonary exercise testing.

**Figure 2:**
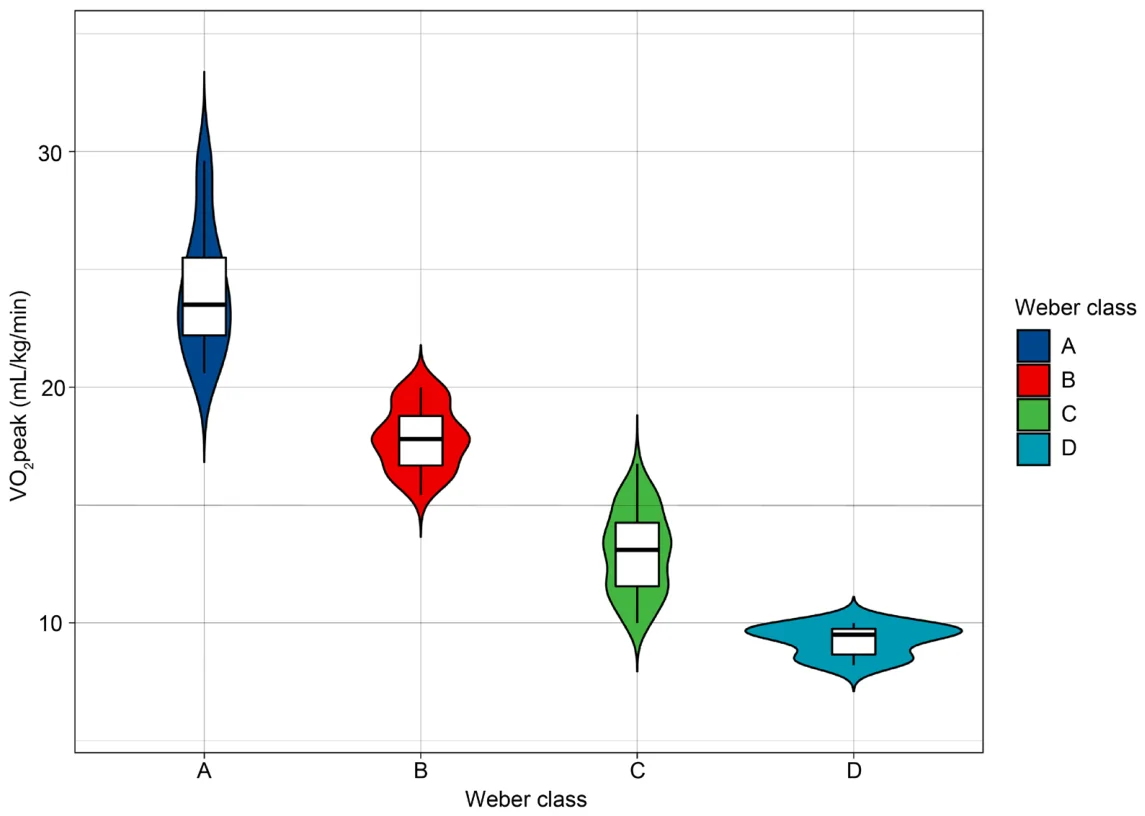
Distribution of CPET VO_2_peak across Weber classification categories. The solid line indicates the threshold required for independent living (15 mL·kg^−1^·min^−1^). Overall, 52.4% (64/122) of participants demonstrated VO_2_peak values below 15 mL·kg^−1^·min^−1^. Abbreviations: CPET = cardiopulmonary exercise testing; VO_2_peak = peak oxygen uptake.

Of the 184 patients who met the inclusion criteria, 122 participants were ultimately included in the final analysis. The reasons for participant exclusion are presented in Figure 1. Detailed baseline characteristics are summarized in Table 1. The mean age of the participants was 56.1 ± 12.8 years. Among the included patients, 43 (35.2%) were female, and 57 (46.7%) were older than 60 years. The median VO_2_peak was 14.8 (11.8, 17.9) mL·kg^−1^·min^−1^, the corresponding mean was 15.50 ± 4.63 mL·kg^−1^·min^−1^, and 52.4% of participants exhibited VO_2_peak values below 15 mL·kg^−1^·min^−1^, which is considered the minimum threshold required for independent living (Figure 2). The mean 6MWD was 321.2 ± 157.2 m. In addition, 8.2% (10/122) of participants received β-blocker therapy, while 49.2% (60/122) were classified as overweight.

### 3.2. Association Between CPET VO_2_peak and 6MWD

Univariate linear regression analysis was performed to evaluate the associations between CPET VO_2_peak and a range of demographic characteristics, clinical variables, lifestyle factors, and 6MWT parameters in patients with cerebral infarction (Table 2). Among these variables, 6MWD demonstrated a significant positive association with VO_2_peak (β = 0.016, *p* = 0.001).

To identify factors independently associated with CPET-derived VO_2_peak, variables that met the threshold of *p* < 0.10 in the univariate analyses were considered candidate predictors for inclusion in the multivariable linear regression analysis. The final model was constructed using a forward selection procedure in which candidate variables were entered sequentially according to the prespecified statistical criterion. After adjustment for age, sex, and BMI, 6MWD remained a significant independent predictor of VO_2_peak (unstandardized coefficient B = 0.010, standardized β = 0.329, *p* < 0.001). Residual diagnostics did not indicate serious violations of the assumptions of linear regression. In the bootstrap sensitivity analysis, the association between 6MWD and VO_2_peak remained significant, with a similar estimate and a BCa 95% confidence interval that did not include zero (B = 0.011, BCa 95% CI: 0.006 to 0.015). In addition, the Physical Activity Score (standardized β = 0.260, *p* < 0.001) and Berg Balance Scale score (standardized β = 0.137, *p* = 0.048) were independently associated with VO_2_peak. Notably, 6MWD demonstrated the largest standardized effect size among the functional and activity-related variables. The complete results of the multivariable regression analyses are presented in Table 3.

**Figure 3:**
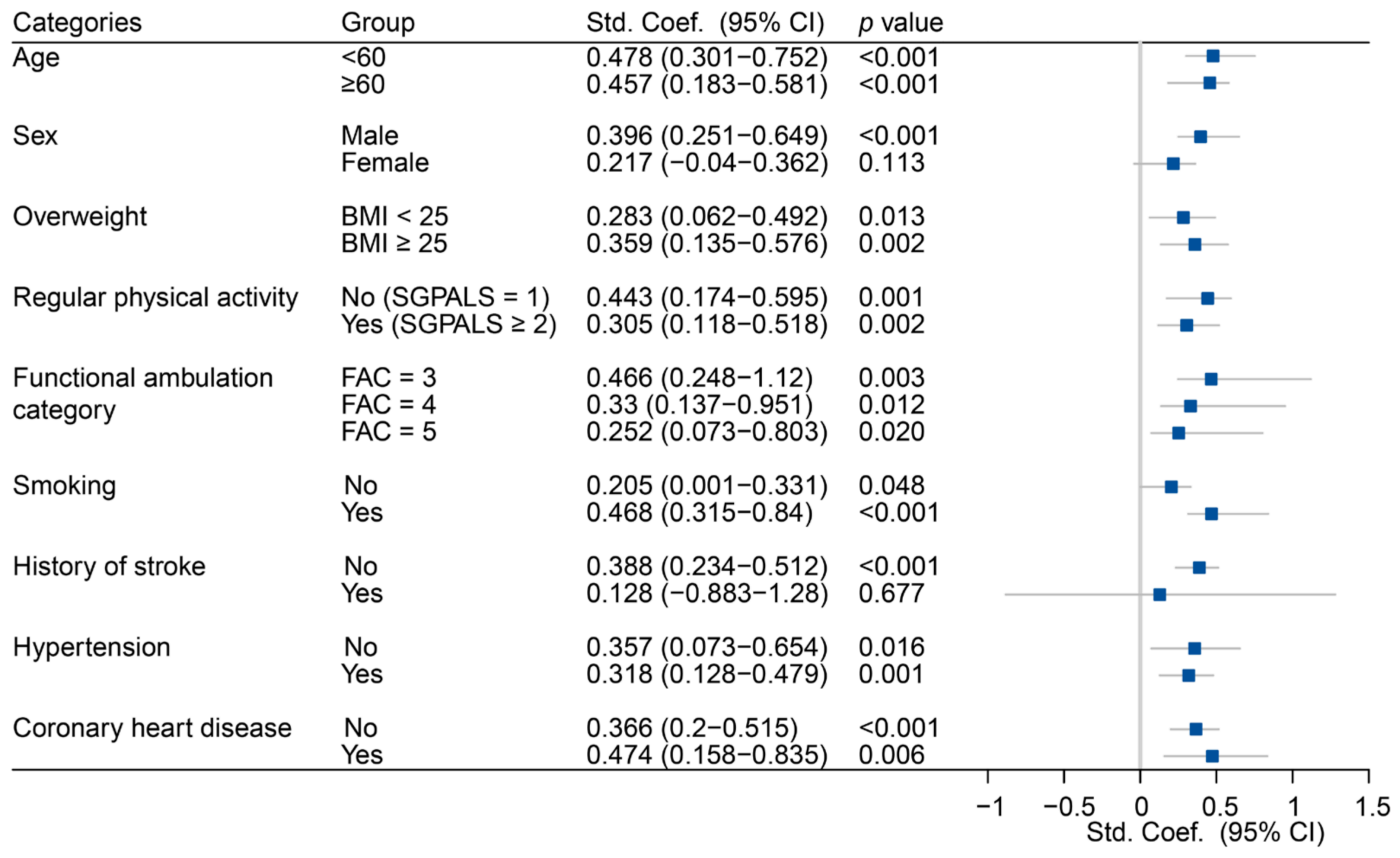
Subgroup analysis of the association between 6MWD and VO_2_peak. Forest plot showing the unadjusted standardized regression coefficients (β) and 95% confidence intervals (CIs) for the association between 6-min walk distance (6MWD) and peak oxygen uptake (VO_2_peak) across clinical subgroups of patients with subacute cerebral infarction. Simple linear regression was performed within each subgroup, with 6MWD as the independent variable and VO_2_peak as the dependent variable. No covariate adjustment was performed due to the limited sample sizes in some subgroups. The vertical line at β = 0 indicates no association. Abbreviations: BMI, body mass index; FAC, Functional Ambulation Category.

Subgroup analyses demonstrated that the positive association between 6MWD and VO_2_peak was generally consistent across most clinical characteristics (Figure 3). The association remained statistically significant (all *p* < 0.05) in the majority of subgroups, including those stratified according to physical activity level, ambulatory function, smoking status, obesity, hypertension, and coronary heart disease. However, the association appeared attenuated and did not reach statistical significance among female participants (*p* = 0.113) and participants with a previous history of stroke (*p* = 0.677).

### 3.3. Indicative Value of the 6MWT for CPET HRpeak

**Figure 4:**
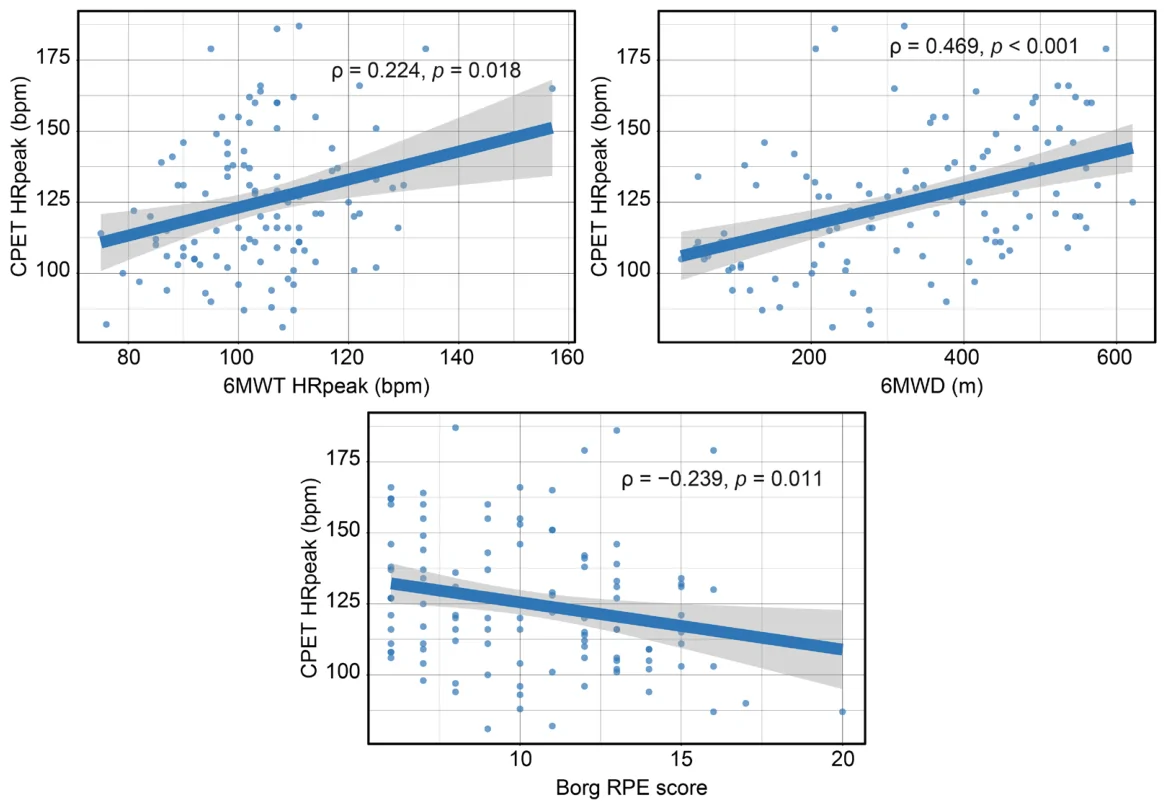
The scatter plots illustrate the associations between CPET HRpeak and 6MWT-derived variables. The blue solid line represents the fitted trend line, and the grey shaded regions represent the 95% confidence interval bands. Abbreviations: CPET HRpeak = peak heart rate during cardiopulmonary exercise testing; 6MWT HRpeak = peak heart rate during the 6-min walk test; 6MWD = distance covered during the 6-min walk test; Borg RPE score = Borg Rating of Perceived Exertion score.

Spearman correlation analyses demonstrated that CPET HRpeak was positively correlated with both 6MWT HRpeak and 6MWD, whereas it was negatively correlated with Borg RPE score (Figure 4).

After adjustment for age, sex, β-blocker use, partial correlation analyses showed that CPET HRpeak was positively correlated with 6MWT HRpeak (ρ = 0.217, *p* = 0.018) and 6MWD (ρ = 0.361, *p* < 0.001), and negatively correlated with Borg RPE score (ρ = −0.243, *p* = 0.015).

### 3.4. Mediation Analysis of 6MWT HRpeak/HRmax-Pred in the Relationship Between 6MWD and VO_2_peak

In the β-blocker-adjusted post hoc exploratory mediation analysis, 6MWT HRpeak/HRmax-pred did not show a significant indirect effect in the association between 6MWD and VO_2_peak (indirect effect = −0.0003, 95% bootstrap CI: −0.0015 to 0.0007). The direct effect of 6MWD on VO_2_peak remained significant after adjustment for β-blocker use (direct effect = 0.0157, 95% CI: 0.0112 to 0.0202, *p* < 0.001). Detailed results are shown in Table 4.

## 4. Discussion

The present study demonstrated that 6MWD was positively associated with VO_2_peak in patients with cerebral infarction within 3 months after the onset of stroke, and this association was not substantially influenced by walking ability, comorbid conditions, BMI, or habitual physical activity level. Furthermore, the relationship between 6MWD and VO_2_peak was independent of the ratio of 6MWT HRpeak to HRmax-pred. In addition, both 6MWD and 6MWT HRpeak were significantly correlated with CPET HRpeak, suggesting that these 6MWT-derived parameters may provide clinically relevant information for the formulation of individualized rehabilitation programs.

Reduced CRF is a common manifestation in patients with cerebral infarction, beginning during the acute stage and frequently persisting throughout the disease course. Nevertheless, systematic CRF evaluation has not been routinely integrated into stroke rehabilitation practice [25], while the supporting research evidence remains insufficient. A systematic review including 81 CRF-related studies involving 3082 patients with stroke reported that the mean VO_2_peak in subacute patients within 6 months after the onset of stroke was 14.34 mL·kg^−1^·min^−1^, corresponding to approximately 50% of the value observed in healthy age-matched individuals. The review additionally demonstrated that CRF exhibited a spontaneous upward trend during the first three months after stroke [2]. In a recent investigation evaluating the effects of aerobic exercise on CRF in patients with cerebral infarction within 3 months after onset [27], the reported mean VO_2_peak was 17.88 ± 5.49 mL·kg^−1^·min^−1^, which was slightly higher than the value of 15.50 ± 4.63 mL·kg^−1^·min^−1^ observed in the present study. In the current cohort of patients with subacute cerebral infarction, Weber classification indicated that only 15% of participants exhibited normal CRF, whereas nearly half were categorized as Weber class C (VO_2_peak 10–16 mL·kg^−1^·min^−1^), reflecting moderate-to-severe CRF impairment. Impaired CRF is frequently associated with reduced social participation and diminished quality of life. A VO_2_peak value of 15 mL·kg^−1^·min^−1^ is generally considered the minimum threshold required for independent living; therefore, this threshold may serve as a clinically meaningful indicator for determining whether CRF impairment compromises an individual’s capacity for independent daily living [28]. Although all participants in the present study were capable of independent ambulation, more than half demonstrated VO_2_peak values below the threshold required for independent living, suggesting a need for individualized intervention strategies.

The present study additionally demonstrated that, after adjustment for age, sex, and BMI, 6MWD remained independently associated with VO_2_peak in patients with subacute cerebral infarction within 3 months after onset. Compared with most previous studies, which primarily focused on the chronic phase of stroke recovery (>6 months after onset) [8,26], the present findings extend CRF assessment into the early “golden window” of functional recovery [29], thereby providing clinically meaningful implications for early intervention strategies. Baert et al. [11] reported that functional walking ability contributed to the multivariable regression model for CRF only at 12 months poststroke. However, the present study demonstrated that 6MWD possessed independent association within the first 3 months after onset. This discrepancy may be attributable to differences in assessment methodology. Specifically, the short-distance gait speed assessment used by Baert et al. [11] primarily reflects instantaneous walking performance, whereas 6MWD, as an indicator of walking endurance, may more accurately reflect daily functional activity capacity. In 2020, Kim et al. [30] similarly reported that 6MWD was correlated with VO_2_peak in patients with cerebral infarction within 3 months after onset; however, in their stepwise regression analysis, 6MWD was replaced by gait speed, muscle strength, and fat mass. This substitution may have resulted from substantial collinearity between gait speed and 6MWD. Supporting this interpretation, Cheng et al. [31] reported a strong positive correlation between gait speed and 6MWT distance in patients with stroke (r = 0.80–0.95). It should additionally be emphasized that the study conducted by Kim et al. [30] required specialized equipment, including an isokinetic dynamometer and a body composition analyzer. In contrast, the present study relied only on basic clinical variables such as age, sex, and BMI, and the 6MWT itself is simple to administer without requiring complex instrumentation, thereby making it more applicable to routine rehabilitation practice. Collectively, these findings suggest that 6MWD is independently associated with CPET-derived VO_2_peak in patients with subacute cerebral infarction. The 6MWT may therefore serve as a practical supplementary tool for detecting impaired CRF when CPET is not readily accessible, although it should not be considered a substitute for CPET in the direct evaluation of cardiorespiratory fitness.

Previous studies have suggested that the degree of neurological impairment may influence the association between 6MWD and CRF [32]. In the subgroup analyses of the present study, this relationship was further evaluated across populations with different clinical characteristics. The findings demonstrated that, irrespective of comorbid conditions, smoking status, obesity, pre-stroke regular exercise habits, or post-stroke ambulatory ability, 6MWD remained significantly and positively associated with VO_2_peak, indicating substantial clinical robustness of this relationship. Among female participants, the association between 6MWD and VO_2_peak did not reach statistical significance (β = 0.217, 95% CI: −0.04 to 0.362, *p* = 0.113), although the direction of the association remained consistent with the overall analysis. This nonsignificant result may reflect the comparatively small number of female participants, who represented 35.2% of the cohort, and the resulting limited statistical power rather than an actual biological or physiological difference. Likewise, among participants with a prior history of stroke, the small proportion of cases (12.3%) reduced statistical power and produced wider confidence intervals. Accordingly, these subgroup results should be interpreted with caution and should not be regarded as evidence that no association exists. Overall, except for several subgroups constrained by limited sample size, 6MWD demonstrated broad applicability as an independent correlate of VO_2_peak in patients with subacute cerebral infarction and consistently reflected impaired CRF. Accordingly, the 6MWT may serve as a practical and clinically accessible alternative method for CRF assessment.

The multivariable linear regression analysis additionally demonstrated that the pre-stroke SGPALS score was positively associated with post-stroke VO_2_peak in patients with cerebral infarction, whereas Berg Balance Scale score exhibited a borderline significant association (*p* = 0.048). The SGPALS has shown considerable utility in multiple clinical and research fields, including chronic disease management, health behavior surveillance, and rehabilitation intervention evaluation [33]. Previous studies have demonstrated that higher levels of pre-stroke physical activity are associated with lower NIHSS scores during the acute phase of cerebral infarction [15], as well as superior motor performance and improved quality of life during subsequent follow-up periods [34]. The present findings further indicate that greater pre-stroke physical activity intensity is positively associated with post-stroke CRF in patients with subacute cerebral infarction, which is consistent with the observations reported by Boss et al. [28]. Several mechanisms may explain this association. First, patients with habitual pre-stroke exercise participation generally possess higher baseline CRF levels [35] and may therefore retain greater CRF reserve capacity after the onset of stroke. Second, pre-stroke physical activity level may additionally reflect long-term health behavior patterns; patients with more active lifestyles may participate more consistently and effectively in rehabilitation training after stroke, thereby facilitating faster CRF recovery. Although the association between Berg Balance Scale score and VO_2_peak only reached borderline statistical significance (*p* = 0.048), this relationship remains clinically relevant. Superior static and dynamic balance function may enable patients to initiate walking rehabilitation earlier and more safely, thereby promoting CRF improvement. As a comprehensive indicator of mobility and exercise confidence, balance function may therefore possess an intrinsic relationship with CRF. Future interventional studies targeting CRF improvement in patients with cerebral infarction may consider incorporating pre-stroke activity history and balance performance into assessment frameworks to facilitate more individualized rehabilitation strategies.

A complete exercise prescription includes the FITT components: frequency, intensity, time, and type. Among these components, exercise intensity can be prescribed and monitored using several indicators, with heart rate being one of the most commonly used. Current guidelines recommend that exercise intensity should be prescribed according to the percentage of HRpeak and the percentage of heart rate reserve, defined as HRpeak minus resting heart rate [36,37]. HRpeak is most accurately obtained through CPET [38]. In 2024, Reis et al. [39] developed an estimation model for CPET HRpeak in patients with chronic stroke, in which 6MWT HRpeak showed substantial explanatory value. In the present study, CPET HRpeak was significantly associated with several 6MWT-derived parameters in patients with subacute cerebral infarction. Specifically, CPET HRpeak was positively correlated with both 6MWT HRpeak and 6MWD, whereas it was negatively correlated with Borg RPE score. These associations remained statistically significant after adjustment for age, sex, and β-blocker use. In this cohort, CPET HRpeak reached approximately 75% of the age-predicted maximal heart rate, which was lower than the normal reference value of 85% [20]. This may reflect an attenuated heart-rate response during symptom-limited maximal exercise. By comparison, 6MWT HRpeak reached approximately 65% of the age-predicted maximal heart rate, consistent with the submaximal nature of the 6MWT. The positive association between 6MWT HRpeak and CPET HRpeak suggests that heart-rate response during the 6MWT may provide useful information on exercise-related heart-rate responsiveness. Patients who showed a greater heart-rate increase during submaximal walking also tended to achieve a higher HRpeak during symptom-limited CPET. Beyond 6MWT HRpeak, the present study further showed that patients with a longer 6MWD tended to achieve a higher CPET HRpeak. To the best of our knowledge, this is the first study to describe this relationship in patients with subacute cerebral infarction. According to the Fick principle described previously, VO_2_peak is determined jointly by cardiac output (heart rate × stroke volume) and arteriovenous oxygen difference. The latter reflects the ability of peripheral tissues, particularly skeletal muscle, to extract and utilize oxygen efficiently. Previous investigations in patients with cerebral infarction have suggested that stroke volume may approach maximal levels during the 6MWT [40], whereas peripheral oxygen utilization is frequently restricted because of impaired skeletal muscle function [12]. Consequently, when higher oxygen demand is required, heart rate elevation may become the principal compensatory mechanism. Because the present study demonstrated that 6MWD independently associated with VO_2_peak, patients with longer walking distances could possess superior overall exercise capacity and therefore require greater heart rate responses during symptom-limited maximal CPET. This may explain the positive correlation observed between 6MWD and CPET HRpeak. Conversely, the inverse association between the Borg RPE score and CPET HRpeak may indicate diminished exercise tolerance or physiological reserve among patients who experience a greater exertional burden during submaximal walking. However, because the Borg RPE score is subjective and may be affected by pain, anxiety, cognitive function, motivation, and communication ability, this interpretation warrants caution. Overall, these findings suggest that the 6MWT may provide information beyond its association with CRF. Walking distance, HRpeak, and perceived exertion during the 6MWT were all related to CPET HRpeak, indicating that the 6MWT could capture multiple aspects of exercise tolerance and heart-rate response. These readily obtainable parameters may provide supplementary information relevant to heart-rate-based exercise intensity assessment when CPET is not readily available.

The ratio of 6MWT HRpeak to HRmax-pred serves as an indicator of chronotropic response during submaximal exercise. In the present study, this parameter did not mediate the association between 6MWD and VO_2_peak in patients with cerebral infarction. This null finding may be attributable to the absence of a significant relationship between 6MWD and 6MWT HRpeak/HRmax-pred. This observation is consistent with the findings reported by Woodward et al. [41], who similarly demonstrated that heart rate response during the 6MWT in patients recovering from stroke was not associated with walking distance, but was instead related to baseline characteristics such as resting heart rate and BMI. Importantly, the mean 6MWD in the present cohort was 321.2 m, and the median FMA score was 96, indicating an overall moderate-to-good functional status. Therefore, it is unlikely that inadequate walking capacity prevented sufficient heart rate mobilization during the 6MWT. The mediation analysis further confirmed that 6MWD was significantly associated with VO_2_peak in patients with subacute cerebral infarction; however, this association was not mediated by 6MWT HRpeak/HRmax-pred. These findings suggest that chronotropic response during submaximal exercise is not the principal physiological mechanism underlying the relationship between walking endurance and CRF in this population.

## 5. Limitations

Several limitations of the present study should be acknowledged. First, because of the cross-sectional study design, causal relationships could not be established, and future longitudinal investigations are required to further clarify these associations. Second, because the mediation analysis was exploratory and performed post hoc, and no a priori power calculation was specifically conducted for this analysis, the study may have had insufficient statistical power to detect small indirect effects. The univariate regression, partial correlation, and subgroup analyses were likewise exploratory and were not adjusted for multiple comparisons; consequently, the possibility of Type I error cannot be excluded. In addition, stroke volume and arteriovenous oxygen difference were not directly measured, thereby limiting the mechanistic interpretation of the findings within the framework of the Fick principle. Finally, all participants had FAC scores ranging from 3 to 5, reflecting relatively preserved ambulatory function. Accordingly, the present findings should not be extrapolated to patients with more pronounced motor impairment or poorer ambulatory function, including those with FAC scores of 1–2.

## 6. Conclusions

The present study demonstrates that the 6MWT possesses important clinical value for assessing CRF in patients with cerebral infarction. Specifically, 6MWD was independently associated with VO_2_peak, and this relationship remained robust across subgroups with different clinical characteristics. Furthermore, both 6MWT HRpeak and walking distance were significantly correlated with CPET HRpeak, suggesting that 6MWT-derived parameters could assist in providing a clinically practical basis for determining appropriate exercise intensity. In addition, 6MWT HRpeak/HRmax-pred did not mediate the relationship between 6MWD and VO_2_peak, indicating that chronotropic response is unlikely to represent the primary physiological link between walking endurance and CRF. Future studies should further investigate alternative physiological mechanisms underlying this association.

## Figures and Tables

**Table 1:** Baseline characteristics of the 122 participants.

	*n* = 122
Demographic Characteristic	
Age (years)	56.1 ± 12.8
Female (%)	43 (35.2)
BMI (kg/m^2^)	25.2 ± 3.4
Patient Characteristics	
Hypertension, *n* (%)	79 (64.8)
Diabetes, *n* (%)	42 (34.4)
Hyperlipemia, *n* (%)	73 (59.8)
Coronary heart disease, *n* (%)	29 (23.78)
Hyperhomocysteinemia, *n* (%)	27 (22.1)
History of cerebrovascular disease, *n* (%)	15 (12.3)
History of smoking, *n* (%)	49 (40.2)
SGPALS	
Level 1, *n* (%)	49 (40.2)
Level 2, *n* (%)	51 (41.8)
Level 3, *n* (%)	21 (17.2)
Level 4, *n* (%)	1 (0.8)
β-blocker, *n* (%)	10 (8.2)
Stroke Features	
TOAST Classification	
Large artery atherosclerosis, *n* (%)	63 (51.6)
Small vessel disease, *n* (%)	29 (23.8)
Cardioembolic, *n* (%)	8 (6.6)
Other determined etiology, *n* (%)	16 (13.1)
Cryptogenic, *n* (%)	6 (4.9)
NIHSS	1 (1, 3)
BBS	52 (47, 56)
FMA	96 (86, 100)
FAC	
Category 3, *n* (%)	41 (33.6)
Category 4, *n* (%)	21 (17.2)
Category 5, *n* (%)	60 (49.2)
CPET	
Onset-to-test time (days)	34 (21.8, 52.5)
VO_2_peak (mL·kg^−1^·min^−1^)	14.8 (11.8, 17.9)
%predicted VO_2_ peak	58.9 ± 15.1
Peak HR (bpm)	120 (106, 137)
%predicted HRmax	75.3 ± 12.1
6MWT	
Onset-to-test time (days)	37 (19, 62)
Distance (m)	321.2 ± 157.2
Resting HR (bpm)	83 ± 10
HR at end of test (bpm)	97 ± 13
Peak HR (bpm)	105 (96, 111)
%predicted HRmax	64.1 ± 10.4
ΔHR (bpm)	24 (19, 33)
Borg RPE score	10 (7, 13)

Data are presented as the mean ± standard deviation, median (Q1, Q3), or *n* (%), as appropriate. Abbreviations: SD = standard deviation; BMI = body mass index; SGPALS = Saltin–Grimby Physical Activity Level Scale; TOAST = Trial of Org 10172 in Acute Stroke Treatment; NIHSS = National Institutes of Health Stroke Scale; BBS = Berg Balance Scale; FMA = Fugl–Meyer Assessment; FAC = Functional Ambulation Category; CPET = cardiopulmonary exercise testing; VO_2_peak = peak oxygen uptake; HR = heart rate; 6MWT = The 6-min walk test; Borg RPE score = Borg Rating of Perceived Exertion score.

**Table 2:** Univariate linear regression analysis for CPET-derived VO_2_peak.

Variables	β	Standard Error	*p* Value	R^2^
Age (years)	−0.18	0.029	<0.001	0.247
Female	−1.466	0.870	0.094	0.023
BMI	−0.261	0.123	0.035	0.036
Hypertension	−1.466	0.870	0.094	0.023
Diabetes	−3.042	0.812	<0.001	0.105
Hyperlipemia	−0.888	1.212	0.465	0.004
Coronary heart disease	−1.619	0.873	0.066	0.028
Hyperhomocysteinemia	−1.515	0.978	0.124	0.020
History of cerebrovascular disease	−0.574	1.280	0.655	0.002
History of smoking	0.169	0.844	0.842	0.000
SGPALS	1.797	0.537	0.001	0.085
β-blocker, *n* (%)	−1.565	1.527	0.307	0.009
NIHSS	−0.811	0.193	<0.001	0.128
BBS	0.121	0.065	0.066	0.028
FMA	0.021	0.021	0.323	0.008
FAC	2.019	0.431	<0.001	0.154
6MWD	0.016	0.002	<0.001	0.278
Borg RPE score	−0.414	0.122	<0.001	0.087

Abbreviations: CPET = cardiopulmonary exercise testing; VO_2_peak = peak oxygen uptake; BMI = body mass index; SGPALS = Saltin–Grimby Physical Activity Level Scale; NIHSS = National Institutes of Health Stroke Scale; BBS = Berg Balance Scale; FMA = Fugl–Meyer Assessment; FAC = Functional Ambulation Category; 6MWD = distance covered during the 6-min walk test; Borg RPE score = Borg Rating of Perceived Exertion score.

**Table 3:** Multivariable linear regression analysis for CPET VO_2_peak.

Variants	Beta	Standard Error	Standard Beta	T Value	*p* Value
Constant	20.950	3.979		5.264	<0.001
Sex	−1.868	0.669	−0.194	−2.792	0.006
Age	−0.141	0.025	−0.391	−5.713	<0.001
BMI	−0.237	0.092	−0.173	−2.585	0.011
SGPALS	1.598	0.401	0.260	3.987	<0.001
BBS	0.099	0.050	0.137	1.997	0.048
6MWD	0.010	0.002	0.329	4.569	<0.001

Abbreviations: CPET = cardiopulmonary exercise testing; VO_2_peak = peak oxygen uptake; BMI = body mass index; SGPALS = Saltin–Grimby Physical Activity Level Scale; BBS = Berg Balance Scale; 6MWD = distance covered during the 6-min walk test.

**Table 4:** Exploratory mediation analysis of 6MWT HRpeak/HRmax-pred in the relationship between 6MWD and VO_2_peak.

Effect	Estimate	95% CI	*p* Value
Indirect	−0.0003	−0.0015 to 0.0007	−
Direct	0.0157	0.0112 to 0.0202	<0.001

Bootstrap confidence intervals were generated using 5000 bootstrap resamples. All analyses were adjusted for β-blocker use. Abbreviations: HRpeak = peak heart rate; HRmax-pred = Age-predicted maximal heart rate; 6MWD = distance covered during the 6-min walk test; VO_2_peak = peak oxygen uptake.

## Data Availability

Due to privacy and ethical concerns, the data supporting the findings of this study are available from the corresponding author upon reasonable request solely for checking the reproducibility of the results.

## Abbreviations

The following abbreviations are used in this manuscript:

6MWT	6-min walk test
CRF	Cardiorespiratory fitness
CPET	Cardiopulmonary exercise testing
VO_2_peak	Peak oxygen uptake
HRpeak	Peak heart rate
6MWD	6-min walk distance
COPD	Chronic obstructive pulmonary disease
SGPALS	Saltin–Grimby Physical Activity Level Scale
NIHSS	National Institutes of Health Stroke Scale
BBS	Berg Balance Scale
FMA	Fugl–Meyer Assessment
FAC	Functional Ambulation Category
RPE	Rating of Perceived Exertion
BMI	Body mass index
